# *Cissampelos sympodialis Eichl* (*Menispermaceae*) leaf extract as a possible novel and safe treatment for psoriasis

**DOI:** 10.1590/S1516-31802009000400011

**Published:** 2009-12-07

**Authors:** Amir Feily, Mohammad Reza Namazi

**Affiliations:** 1 MD. Resident in Dermatology, Department of Dermatology, Jondishapur University of Medical Sciences, Ahvaz, Iran.; 2 MD. Assistant professor in the Dermatology, Medicinal and Natural Product Chemistry Research Center and Dermatology Department, Shiraz University of Medical Sciences, Shiraz, Iran.

Psoriasis is an inflammatory disorder characterized by a T helper cell type 1 cytokine pattern. Interferon-gamma (IFN-g), tumor necrosis factor-alpha (TNF-a), interleukin-2 (IL-2) and interleukin-1 (IL-1) are predominantly expressed in this disorder. This immunological pathway is stimulated by interleukin-12 (IL-12) released from activated dendritic cells and by exposure of T cells to type 1 cytokines during maturation.^1^ Systemic administration of T helper 2 (Th2) cytokines such as interleukin-4 (IL-4) or interleukin-10 (IL-10) neutralizes the T helper 1 (Th1) bias and improves psoriasis.^2^^,^^3^ TNF-a is believed to be a key pro-inflammatory cytokine involved in psoriasis pathogenesis, and anti-TNF blockers initially developed for rheumatoid arthritis therapy have dramatically decreased the clinical activity of psoriasis lesions.^4^ In psoriatic epidermis, the level of cyclic adenosine monophosphate (cAMP) decreases. It has been reported that beta-blockers may exacerbate psoriatic plaques through decreasing the concentration of intracellular cAMP. Topical caffeine, which is a methylxanthine, induces a higher concentration of intracellular cAMP and hence is an effective treatment for psoriasis.^5^ Nitric oxide synthesis is increased in psoriasis and a role for nitric oxide in the development of psoriasis has been suggested by some researchers.^1^

Leaves from *Cissampelos sympodialis Eichl* (*Menispermaceae*) were collected in João Pessoa, State of Paraíba, Brazil, in January 1998. Infusions of *C. sympodialis* roots are popularly used in northeastern Brazil for treating asthma, bronchitis and rheumatism, among other inflammatory diseases. The immunomodulatory effect of the aqueous fraction of the ethanolic extract of the leaves (AFL) of *C. sympodialis* has been described. This effect was associated with inhibition of IL-2 production and increased production of both IL-10 and IL-4. AFL thus has a potent anti-inflammatory effect through downregulation of inflammatory cytokines.^6^

It has been shown that AFL inhibits cyclic nucleotide phosphodiesterase activity and increases cAMP levels in intact smooth cell cultures, pig bronchoalveolar leukocytes and murine B cells. Recent findings have shown that cAMP mimetic or activating reagents inhibit secretion of both TNF-alpha and IL-12 by activated peritoneal macrophages. This inhibitory effect has been shown to be mediated by increased IL-10 secretion. It has also been shown that AFL increased *Trypanosoma cruzi* growth through reduction of NO production. This effect may be mediated by an autocrine mechanism that depends on secretion of IL-10 by macrophages.^6^

Taking all the above facts together, given the potent effect of AFL towards decreasing the production of NO and inflammatory cytokines involved in the pathogenesis of psoriasis and increasing the production of anti-inflammatory cytokines (which are known to ameliorate this disease), we hereby suggest that *Cissampelos sympodialis Eichl* (*Menispermaceae*) leaf extract could be a novel and safe addition to the antipsoriatic weaponry.
